# Degradation and Transformation Mechanisms of *Zanthoxylum* Alkylamides Exposed to UVB Light

**DOI:** 10.3390/foods12244392

**Published:** 2023-12-06

**Authors:** Rui Wang, Chaolong Rao, Qiuyan Liu, Xiong Liu

**Affiliations:** 1 College of Food Science, Southwest University, Chongqing 400715, China; 2 State Key Laboratory of Southwestern Chinese Medicine Resources, School of Public Health, Chengdu University of Traditional Chinese Medicine, Chengdu 611137, China; 3 Collaborative Innovation Center for Child Nutrition and Health Development, Chongqing University of Education, Chongqing 400067, China

**Keywords:** *Zanthoxylum* alkylamides, UVB light, degradation, transformation, mechanism

## Abstract

*Zanthoxylum* oleoresin, a concentrated extract derived from *Zanthoxylum bungeanum*, is rich in non-volatile, intensely flavorful substances and amide compounds, such as hydroxy-α-sanshool, hydroxy-β-sanshool, and hydroxy-ε-sanshool. The production process of *Zanthoxylum* oleoresin remains unstandardized, and there is still a lack of research on the precise classification and quantification of its key chemical constituents, as well as the stability of these compounds when produced using different extraction methods. This study utilized preparative liquid chromatography to extract and purify amide compounds from *Zanthoxylum* oleoresin, successfully isolating three sanshools: hydroxy-α-sanshool, hydroxy-β-sanshool, and hydroxy-ε-sanshool. The stability of three these sanshools under UVB irradiation in different solvents was explored in UVB-simulated sunlight conditions to investigate the degradation or transformation mechanism of *Zanthoxylum* alkylamides under UVB irradiation. The findings indicate a rapid decrease in the hydroxy-α-sanshool content under UVB ultraviolet light, aligning with the second-order kinetics. This study revealed alterations in the contents of hydroxy-α-sanshool, hydroxy-β-sanshool, and hydroxy-ε-sanshool and the formation of a new compound following exposure to UVB light. This new compound, along with the three sanshools, possesses a uniform *m*/*z* 264 and shares similar chemical structures. Further analysis also uncovered that these compounds are capable of undergoing isomerization reactions under UVB irradiation. This demonstrates that UVB irradiation of certain intensities can modify the concentrations and chemical structures of these *Zanthoxylum* alkylamides. These insights offer crucial guidance for future studies on the processing and preservation of *Zanthoxylum* alkylamides and their derivatives.

## 1. Introduction

*Zanthoxylum bungeanum*, a spice plant of the genus *Zanthoxylum* L. in the family Rutaceae with about 250 species, is mainly distributed in the tropical and subtropical regions of Asia, America, Africa, and Oceania [1]. In China, *Z. bungeanum* is mainly distributed south of the Yangtze River and in the southwestern provinces, distinguishing between red and green *Z. bungeanum* [2]. With its unique numbing taste and spicy flavor, *Z. bungeanum* is widely used in hot pot and other Sichuan dishes [3]. It is one of the “eight spices” people use in their day-to-day life [4].

*Z. bungeanum* mainly contains chemical constituents such as amides, volatile oils, and alkaloids [5,6,7], and its fruits, roots, branches, and leaves can be used as medicinal herbs. *Zanthoxylum* alkylamides, a class of long-chain polyunsaturated amides extracted from *Z. bungeanum*, have been isolated, and more than 25 *Zanthoxylum* amides have been identified, mainly including α-sanshool, β-sanshool, γ-sanshool, δ-sanshool, and their amino part with hydroxylated congeners [8,9,10]. With strong stimulating properties, they are the material basis of the numbing taste of *Z. bungeanum* [11]. Recent studies have shown that the numbing components exhibit anesthetic, analgesic, antioxidant, and anticancer properties [4,12,13].

As we all know, ultraviolet light from the sun is an inescapable part of the environment on the earth’s surface. Generally, ultraviolet radiation is classified into ultraviolet radiation A (UVA), ultraviolet radiation B (UVB), and ultraviolet radiation C (UVC), which correspond to wavelength ranges of 400–315 nm, 315–280 nm, and 280–200 nm, respectively. UVC radiation is entirely absorbed by the earth’s atmosphere, allowing primarily UVA and a lesser proportion of UVB to reach the earth’s surface [14]. Contrary to the minimal harm of UVA radiation, UVB, although a minor component of solar radiation, causes the most substantial environmental damage to the earth. Recent years have witnessed an escalation in UVB radiation reaching the earth’s surface, attributed to the degradation of the ozone layer [15]. This increase in ultraviolet radiation exerts significant effects on biological cells. It can also modify the vitamins, fatty acids [16], and other substances in plants such as *Zanthoxylum bungeanum*. *Zanthoxylum armatum*, another spice plant of the genus *Zanthoxylum* L., primarily comprises hydroxy-sanshool compounds such as hydroxy-ε-sanshool, hydroxy-α-sanshool, hydroxy-β-sanshool, and hydroxy-γ-sanshool, with hydroxy-α-sanshool being the predominant compound. The harvest of this plant occurs before full maturity, leading to significant variations in quality at different stages of ripeness. The concentration of its characteristic numbing agents initially increases with seed maturation, reaching a peak in the intermediate stage of ripeness, and subsequently diminishes in the later stages of maturity [17]. Currently, there is no established research investigating the potential correlation between level changes in the alkylamides of *Zanthoxylum bungeanum* during its ripening process and UVB radiation exposure.

*Zanthoxylum* oleoresin, a concentrated extract derived from *Zanthoxylum bungeanum*, is rich in non-volatile, intensely flavorful substances and amide compounds like hydroxy-α-sanshool, hydroxy-β-sanshool, and hydroxy-ε-sanshool. The production process of *Zanthoxylum* oleoresin remains unstandardized, with a notable gap in research regarding the precise classification and quantification of its key chemical constituents, as well as the stability of these compounds when produced using different extraction methods. The chemical structure of *Zanthoxylum* alkylamides usually contains two or more conjugated double bonds, resulting in the instability of piperine amide crystals. The content of a hydroxy-α-sanshool aqueous solution (0.2 mg/mL, pH 7.0) was reduced by 50% after it was left at room temperature for 4 weeks. The *Zanthoxylum* alkylamides were also susceptible to degradation at high temperatures. The content of an 80% ethanolic solution containing 200 ppm of hydroxy-α-sanshool was also rapidly reduced when placed under ultraviolet (UV) light [18]. Ultraviolet light with a wavelength of 254 nm belongs to the UVC spectrum, which is almost non-existent on the earth’s surface due to its weak penetration ability. As a non-thermal disinfection technology, UVC is commonly used in fruit and vegetable preservation, and for disinfecting surfaces and water [19]. Consequently, studying hydroxy-sanshool compounds under UVC irradiation does not accurately represent their property changes when exposed to sunlight. Additionally, the *Zanthoxylum* alkylamides were not stable enough under acidic conditions [20]. Thus, it was presumed that the poor stability of *Zanthoxylum* alkylamides of natural origin limited their wide application.

At present, the research on the stability and degradation mechanism of *Zanthoxylum* alkylamides is not extensive, limiting their application. Therefore, an in-depth study on the changes in these compounds under UVB irradiation can help explore the processing and storage of *Z. bungeanum*, which is essential for the scientific and rational use of *Zanthoxylum* and its related products.

## 2. Materials and Methods

### 2.1. Chemicals and Experimental Material

Methanol (chromatographic grade) and acetonitrile (chromatographic grade) were purchased from Aladdin Biochemical Technology Co., Ltd. (Shanghai, China). The chromatographic silica gel GF254 was purchased from Haiyang Chemical Co., Ltd. (Qingdao, China). Formic acid, ammonium acetate, ether, and petroleum ether were purchased from Ke Long Chemical Reagent Co., Ltd. (Chengdu, China). Methanol, ethanol, and dichloromethane were purchased from Chuandong Chemical Co., Ltd. (Chongqing, China). *Zanthoxylum* oleoresin was purchased from Xuemailong Food Spice Co., Ltd. (Zhengzhou, China).

### 2.2. Extraction of Zanthoxylum Alkylamides

A total of 40 g of chromatographic silica gel powder was weighed in a clean and dry beaker. The powder was then placed in an electric drying oven and activated at 120 °C for 60 min. After activation, it was transferred to a glass desiccator and allowed to cool to room temperature. Next, 20 g of *Zanthoxylum* oleoresin was mixed with the activated silica gel powder. To this mixture, 200 mL of anhydrous ether was added and mixed continuously for 2 h. The resulting mixture was filtered under a vacuum, draining off the excess ether. Then, 600 mL of methanol was added to the residue and thoroughly mixed. The mixture was further extracted in a water bath at 55 °C for 6 h. Following extraction, the mixture was filtered under a vacuum, the filtrate was collected and evaporated on a rotary evaporator. To the concentrated extract, 100 mL of petroleum ether was added and vortexed. The resulting supernatant was transferred into a glass filament/mouth flask, sealed, and placed at −20 °C for freezing and crystallization to obtain the crude extract of *Zanthoxylum* alkylamides [21].

### 2.3. Isolation and Purification of Zanthoxylum Alkylamides

The crude extracts of *Zanthoxylum* alkylamides were transferred to a test tube, and petroleum ether was evaporated using nitrogen blowing. The methanolic solution of the crude extracts of *Zanthoxylum* alkylamides was resolubilized by adding an appropriate amount of methanol to produce a methanolic solution of *Zanthoxylum* alkylamides. This methanolic solution was used for separating and purifying the extracts with a Hanbang NS4000 preparative liquid chromatograph (Hanbang Sci. & Tech. Co., Ltd., Huai’an, China) [8]. CSTCHROM C18 preparative column (20 × 250 mm^2^, 10 μm; Kesheng Experimental Equipment Co., Ltd., Suzhou, China) was used; the mobile phase was methanol/water (55:45). The flow rate was 10 mL/min, and the detection wavelength was 254 nm. The fractions corresponding to the peak times were combined, and methanol was removed at 60 °C using a rotary evaporator. Then, the vials were sealed with a sealing film and placed in a refrigerator at −80 °C for 24 h to freeze. Then, the frozen spin vials were placed in a vacuum freeze dryer to produce the isolated and purified *Zanthoxylum* alkylamides and their monomeric substances with vacuum freeze drying.

### 2.4. Detection of Zanthoxylum Alkylamides

#### 2.4.1. Detection of *Zanthoxylum* Alkylamides Using High-Performance Liquid Chromatography–Mass Spectrometry (HPLC-MS)

The purified *Zanthoxylum* alkylamides were identified with HPLC-MS. The alkylamides (0.5 mg/mL in methanol) were analyzed using an AB 6600+ system equipped with a diode array detector (DAD) and an AB Sciex Triple TOF 6600+ mass spectrometer system (AB Sciex, Darmstadt, Germany). The samples were applied to a Luna Omega Polar C18 column (100 × 2.1 mm^2^, 3 μm; Phenomenex Ltd., Tianjin, China) and eluted using a mobile phase composed of acetonitrile and water (30:70) at a flow rate of 0.3 mL/min. The wavelength for detection was set at 254 nm. The run time was 20 min, and the injection volume was 1 μL. The column temperature was maintained at 40 °C. The parameters of the mass spectrometer system were set as follows: nitrogen pressure, 35 psi; turbo ion spray probe, 500 °C; ion spray voltage, 5500 V, positive mode; declustering potential, 450 V; entrance potential, 80 V; and collision energy, 35 V. The ions were scanned in the range of *m*/*z* 100–1500. The mass spectrometry results thus obtained were used to determine the alkylamide composition against the available references.

#### 2.4.2. Determining *Zanthoxylum* Alkylamides Using HPLC Method

The samples were analyzed using a Thermo Fisher UltiMate 3000 HPLC system equipped with a DAD (Thermo Fisher Scientific Inc., Waltham, MA, USA). The samples were applied to an Accucore C18 column (150 × 4.6 mm^2^, 2.6 μm; Thermo Fisher Scientific Inc., Waltham, MA, USA) with a column temperature of 35 °C, and a flow rate of 0.5 mL/min. The detection wavelength of DAD was 254 nm. A gradient mobile phase composed of methanol (A) and water (B) was used for the HPLC analysis. The linear gradient program of phase A started from 70%, increased to 90% from 0 to 12 min, and further increased to 100% at 15 min.

#### 2.4.3. HPLC Methodology in Detecting *Zanthoxylum* Alkylamides

##### Linearity and Correlation Coefficient

The purified *Zanthoxylum* alkylamides were prepared into a stock solution of 2.0 mg/mL with methanol. The stock solution was diluted into working solutions of 0, 0.2, 0.4, 0.6, 0.8, and 1.0 mg/mL with methanol, filtered through a 0.22-μm microporous membrane, and detected with the HPLC method described in Section 2.4.2. The sample injection volume was 5 μL. A standard curve was generated by plotting the peak area of each substance on the vertical coordinate (*Y*) and the corresponding concentration on the substance as the horizontal coordinate (*X*). The linear regression equation and correlation coefficient (*R*^2^) were calculated.

##### Repeatability and Precision

The mixed standards of *Zanthoxylum* alkylamides at three concentrations of 1.0, 0.7, and 0.36 mg/mL in methanol were tested with the HPLC method described in Section 2.4.2. Five consecutive injections were performed in a single day (intraday) and for five consecutive days during the same time period (interday). Each sample was repeated three times with an injection volume of 5 μL to investigate the intraday and interday reproducibility of each substance and the precision of the instrument.

##### Recovery

A total of 0.1 mg/mL of *Zanthoxylum* alkylamide specimen was prepared with methanol, and an appropriate amount of *Zanthoxylum* alkylamide mixed standard was added precisely so that the concentration of the mixed standard specimen after spiking was 0.15, 0.3, and 0.5 mg/mL, respectively. The mixed standard specimen was tested with the HPLC method described in Section 2.4.2. The spiking test was repeated five times for each sample, and the spiked recovery of each substance was calculated.

### 2.5. UVB Photodegradation Kinetics of Zanthoxylum Alkylamides

The methanolic solution, anhydrous ethanol solution, 50% methanol solution, 50% ethanol solution, and soybean oil samples were prepared with 0.3 mg/mL of *Zanthoxylum* alkylamides. Then, 5 mL of each sample was drawn into a 5-mL quartz cuvette with a stopper, filled with nitrogen, capped and sealed, and irradiated at a distance of 10 cm under UVB lamp with a wavelength of 302 nm (Guanhongya Optoelectronic Technology Co., Ltd., Shenzhen, China) at room temperature in order to accelerate the experiment [22]. The UV intensity of the UVB light source was measured with a UV irradiation meter (Suruide Technology Co., Ltd., Shenzhen, China) and was found to be 180 μW/cm^2^. Then, 1 mL was sampled after 0, 2, 4, 6, 8, and 10 h of HPLC analysis. The soybean oil samples were placed in volumetric flasks, heated in a water bath at 50 °C for 2 h (with shaking every 0.5 h during this period), cooled to room temperature, and fixed to 10 mL with chromatographic-grade methanol. Then, 1.5 mL of the samples was mixed and placed in a centrifuge tube and centrifuged at 12,000 rpm for 10 min. The supernatant was taken and filtered through a 0.22 µm membrane for all samples except soybean oil samples. The rest of the samples, except for the soybean oil samples, were filtered directly through 0.22 µm membranes. All these samples were assayed with the HPLC method described in Section 2.4.2. The UV degradation kinetics were studied according to the changes in the content of *Zanthoxylum* alkylamides in different solvents after UVB irradiation [23,24,25].

### 2.6. Analysis of UVB Light Degradation products of Zanthoxylum Alkylamides and Degradation Pathway

#### 2.6.1. HPLC Analysis of the UVB Degradation Products of *Zanthoxylum* Alkylamides

A total of 10 mg of hydroxy-ε-sanshool, hydroxy-α-sanshool, and hydroxy-β-sanshool were weighed accurately, dissolved in methanol, and fixed to 20 mL to make a sample solution for use. Then, 5.0 mL of each solution was pipetted into a 5.0 mL quartz cuvette with a stopper, sealed with a sealing film, and placed under UVB light for 30 min. Next, 1.0 mL of each sample solution was pipetted into a disposable syringe, filtered with a 0.22 μm microporous membrane, and analyzed with the HPLC method described in Section 2.4.2. The possible degradation products of *Zanthoxylum* alkylamides after UV degradation were determined by comparing the chromatographic peaks of the three sanshools before and after UVB illumination.

#### 2.6.2. Preparative Liquid Chromatographic Separation and Purification of the UVB Degradation Products of *Zanthoxylum* Alkylamides

The mixture of *Zanthoxylum* alkylamides obtained earlier (Section 2.6.1) after UVB photodegradation was subjected to HPLC analysis using a Thermo Fisher UltiMate 3000 HPLC system (Thermo Fisher Scientific Inc., Waltham, MA, USA). The HPLC analysis was performed on an UltiMate AQ-C18 column (4.6 × 250 mm^2^, 5 μm particle size packing) with a mobile-phase flow rate of 0.9 mL/min at 35 °C. The DAD detection wavelength was 254 nm. A gradient mobile phase composed of methanol (phase A) and water (phase B) was used for the HPLC analysis. The linear gradient program of phase A started from 10%, and increased to 100% from 0 to 100 min. The separation was performed with preparative liquid chromatography based on the degradation product peaks determined using HPLC analysis. The elution conditions of the preparative liquid phase were as follows: CSTCHROM preparative column (20 × 250 mm^2^, 10 μm); flow rate: 10 mL/min; detection wavelength: 254 nm; and injection volume: 5 mL. The mobile phase was eluted with 55% aqueous methanol solution in equal measure. The fractions were collected according to the UV degradation product peaks of the identified *Zanthoxylum* alkylamides.

#### 2.6.3. ^1^H Nuclear Magnetic Resonance Spectroscopy (^1^H NMR) of the UVB-Light Degradation Products of *Zanthoxylum* Alkylamides

A certain volume of the distillate of UVB photodegradation products of *Zanthoxylum* alkylamides was taken, transferred into a clean and dry 1 L spin flask, and concentrated with a rotary evaporator in a water bath at 60 °C to evaporate the methanol as completely as possible. In a fume hood, the concentrated solution obtained with rotary evaporation was transferred to a 500 mL clean and dry separatory funnel, and an appropriate amount of dichloromethane was added for extraction. After dichloromethane was thoroughly mixed with the concentrate, the partition funnel was left to stand, and the mixture was stratified. The collected lower layer was transferred to another clean and dry spin vial, and dichloromethane was allowed to evaporate by rotation at 40 °C in a water bath. Dichloromethane was allowed to evaporate for 5 min to fully remove dichloromethane. After the spin evaporation, the vial was removed, and 0.6 mL of deuterated chloroform solution was added to the vial to fully dissolve the substance to be measured inside the vial. However, the deuterated chloroform solution in the spin vial was transferred to the NMR tube for ^1^H NMR analysis.

The three monomeric components of *Zanthoxylum* alkylamides, including hydroxy-ε-sanshool, hydroxy-α-sanshool, and hydroxy-β-sanshool, were weighed precisely 10 mg of each, dissolved in 0.6 mL of deuterated chloroform solution, and transferred to an NMR tube for ^1^H NMR analysis [8].

#### 2.6.4. UVB Degradation Pathways of *Zanthoxylum* Alkylamides

The possible degradation pathways of *Zanthoxylum* alkylamides after UVB irradiation were inferred by analyzing the HPLC chromatograms, mass spectra, and NMR H-spectra of hydroxy-ε-sanshool, hydroxy-α-sanshool, hydroxy-β-sanshool, and UVB degradation products [26].

### 2.7. Data Processing

Unless otherwise noted, the data were obtained from three parallel determinations. Linear regression analysis was conducted using Microsoft Excel 2013 and GraphPad Prism 7 software, and graphical editing was performed using GraphPad Prism 7 and Microsoft PowerPoint 2013 software.

## 3. Results and Discussion

### 3.1. Liquid Chromatogram for Preparing Zanthoxylum Alkylamides

**Figure 1:**
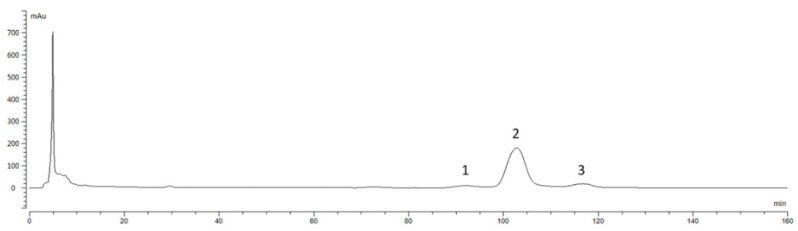
Preparation liquid chromatogram of *Zanthoxylum* alkylamides (1. Isolate 1; 2. Isolate 2; 3. Isolate 3).

The preparative liquid chromatogram of *Zanthoxylum* alkylamides in Figure 1 shows that the peaks appearing in 0–10 min were mainly for the more polar substances, whereas the peaks for the relatively less polar *Zanthoxylum* alkylamides mainly appeared in the time range of 60–85 min. The chromatogram shows that the *Zanthoxylum* alkylamides mainly consisted of isolates 1, 2, and 3. The crude extracts of *Zanthoxylum* alkylamides were thus separated and purified by repeated injection and fraction collection operations in the preparative liquid phase for subsequent relevant experimental studies.

### 3.2. Component Analysis of Zanthoxylum Alkylamides

**Figure 2:**
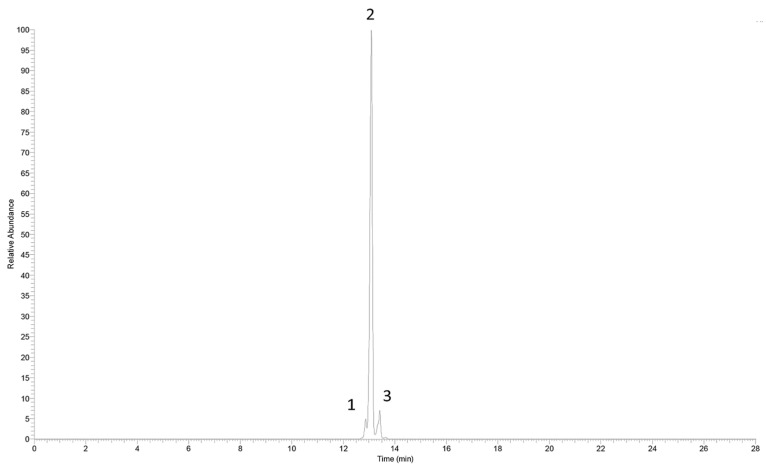
LC-MS total ion current chromatogram of *Zanthoxylum* alkylamides (1. Isolate 1; 2. Isolate 2; 3. Isolate 3).

**Figure 3:**
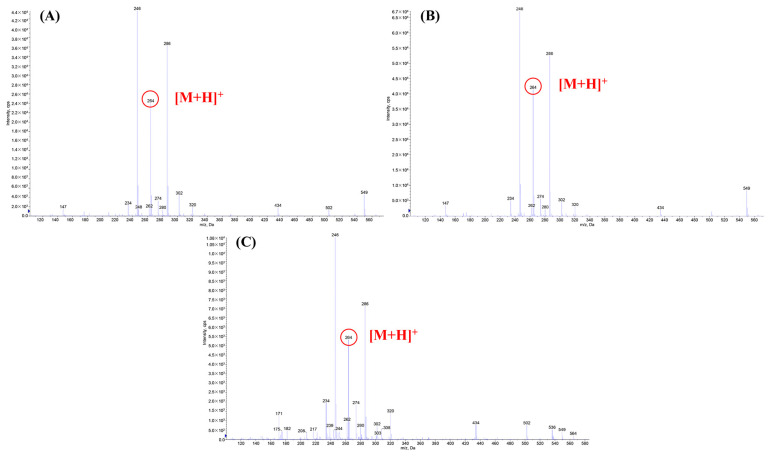
Mass spectra of *Zanthoxylum* alkylamides ((**A**) Isolate 1; (**B**) Isolate 2; (**C**) Isolate 3).

The *Zanthoxylum* alkylamides obtained from the preparation and purification processes described in Section 2.3 were analyzed with the LC-MS detection method elaborated in Section 2.4.1 to determine the composition of the *Zanthoxylum* alkylamides. The LC-MS total ion flow diagrams of the *Zanthoxylum* alkylamides in Figure 2 show that the purified *Zanthoxylum* alkylamides had three mass spectral peaks, corresponding to isolates 1, 2, and 3 of the aforementioned *Z. bungeanum* sesame substances, respectively. The mass spectra depicted in Figure 3 shows that all three *Zanthoxylum* alkylamides had ion peaks with *m*/*z* 264. A comparative analysis of the findings [14] showed that isolates 1, 2, and 3 were hydroxy-ε-sanshool, hydroxy-α-sanshool, and hydroxy-β-sanshool, respectively. The aforementioned results showed that the *Zanthoxylum* alkylamides obtained with preparative liquid chromatography were mainly composed of the aforementioned three sanshools, all of which had a molecular weight of 263 and were isomers of each other.

### 3.3. High-Performance Liquid Chromatograms of Zanthoxylum Alkylamides

**Figure 4:**
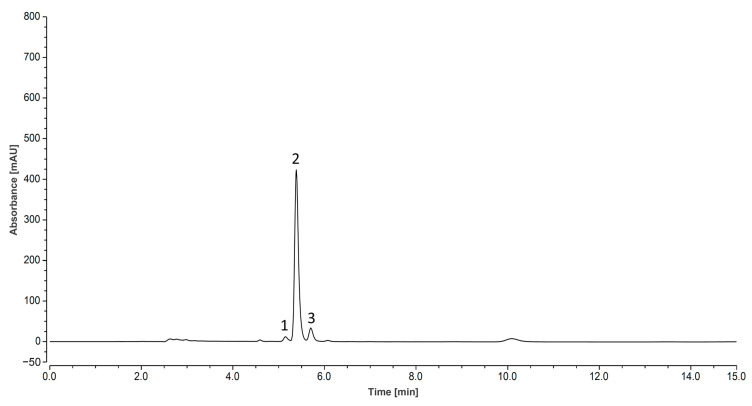
Liquid chromatogram of *Zanthoxylum* alkylamides (1. hydroxy-ε-sanshool; 2. hydroxy-α-sanshool; 3. hydroxy-β-sanshool).

High-performance liquid chromatography (HPLC) is an analytical technique used to separate, identify, or quantify each component in a mixture. In the research related to the *Zanthoxylum* alkylamides, the HPLC method is usually used for analysis and detection [27]. Studies have shown that the *Zanthoxylum* alkylamides has the largest peak area at 254 nm [28]. Moreover, at a column temperature of 35 °C, several types of *Zanthoxylum* alkylamides have better separation effects [29]. Therefore, in this study, the column temperature was 35 °C, and the detection wavelength was 254 nm. As shown with the high-performance liquid chromatograms of *Zanthoxylum* alkylamides in Figure 4, the separation of the three *Zanthoxylum* alkylamides, hydroxy-ε-sanshool, hydroxy-α-sanshool, and hydroxy-β-sanshool, was good under the established liquid-phase conditions. Also, the purity of the *Zanthoxylum* alkylamides was high, as detected using the area normalization method. The content of the three sanshools was 5.9%, 87.2%, and 6.9%, respectively. The aforementioned results indicated that the liquid-phase conditions could be used for determining the content of the three sanshools.

### 3.4. Methodological Testing of the HPLC Method for Detecting Zanthoxylum Alkylamides

#### 3.4.1. Linear

Table 1 shows that the standard curve equation of *Zanthoxylum* alkylamides was as follows: *Y* = 120.99*X* − 4.3988, with a linear range of 0–1.0 mg/mL. The correlation coefficient of hydroxy-ε-sanshool was calculated as follows: *Y* = 49.776*X* + 0.1358, with a linear range of 0–0.06 mg/mL. The standard curve equation of hydroxy-α-sanshool was as follows: *Y* = 124.7*X* − 3.9475, with a linear range of 0–0.9 mg/mL. The standard curve equation of hydroxy-β-sanshool was as follows: *Y* = 129.64*X* − 0.2426, with a linear range of 0–0.07 mg/mL. The correlation coefficients of the standard curve equations for the aforementioned substances were all greater than 0.986, indicating that that the linearity for each sanshool was reliable, and the standard curve equation could be used for the subsequent analysis of the content of *Zanthoxylum* alkylamides [30].

#### 3.4.2. Repeatability and Precision

As depicted in Table 2, the relative standard deviations (RSDs) of different contents of *Zanthoxylum* alkylamides and three sanshools were in the range of 0.04–1.3% under the established HPLC detection conditions, indicating that the instrument used for HPLC analysis had good precision, and could be used for these experiments [31].

As depicted in Table 3, the RSDs of intraday reproducibility were in the range of 0.03–5.25%, whereas the RSDs of interday reproducibility were in the range of 0.27–3.48% for different contents of *Zanthoxylum* alkylamides and the three sanshools under the established HPLC detection conditions. The aforementioned results indicated that the content of *Zanthoxylum* alkylamides and sanshools did not change significantly, and the stability and the interday and intraday reproducibility of HPLC were good.

#### 3.4.3. Recovery

As shown in Table 4, the recoveries of the different samples that spiked under the same chromatographic conditions were in the range of 89.8%–116.4%. This indicated that the established HPLC method for detecting *Zanthoxylum* alkylamides had good recoveries and that the assay had good accuracy. From all the results of our methodology, it can be concluded that the HPLC method is reliable and effective, and can be used for the detection of *Zanthoxylum* alkylamides [32].

### 3.5. Degradation Kinetics of Zanthoxylum Alkylamides under UVB Irradiation

**Figure 5:**
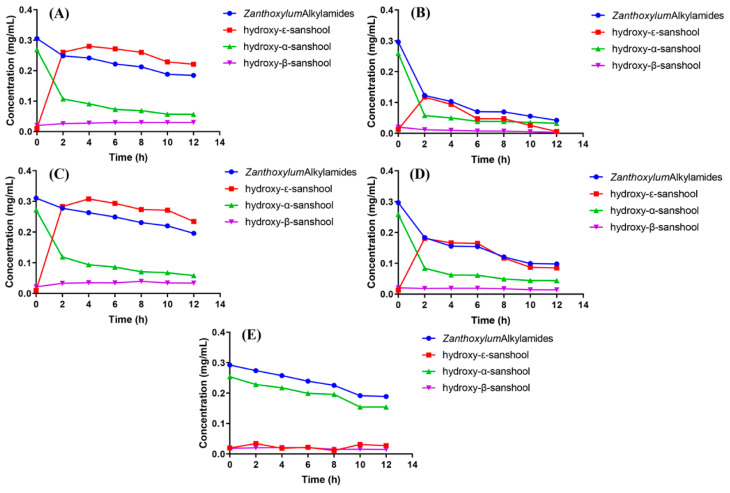
Content of *Zanthoxylum* alkylamides under UVB irradiation ((**A**) methanol; (**B**) 50% methanol; (**C**) ethanol; (**D**) 50% ethanol; (**E**) oil).

As shown in Figure 5, the total amount of *Zanthoxylum* alkylamides and the content of three types of sanshools, namely hydroxy-ε-sanshool, hydroxy-α-sanshool, and hydroxy-β-sanshool, in different solvents had more obvious changes under this UVB irradiation condition. The total amount of *Zanthoxylum* alkylamides and hydroxy-α-sanshool in soybean oil showed a decreasing trend with increasing time of UVB irradiation. However, the contents of hydroxy-ε-sanshool and hydroxy-β-sanshool showed a less obvious change with increasing time of UVB irradiation. The total amount of *Zanthoxylum* alkylamides in four solvents, methanol, ethanol, 50% methanol, and 50% ethanol, showed a decreasing trend. Compared with ultraviolet radiation A (UVA) and UVB irradiation, the effect of UVB on *Zanthoxylum* alkylamides was more obvious. Also, the content of hydroxy-α-sanshool showed a constant decreasing trend, whereas the content of hydroxy-ε-sanshool showed an obvious trend of first increasing and then decreasing. Among the three types of sanshools, the trend of hydroxy-β-sanshool was relatively insignificant.

**Figure 6:**
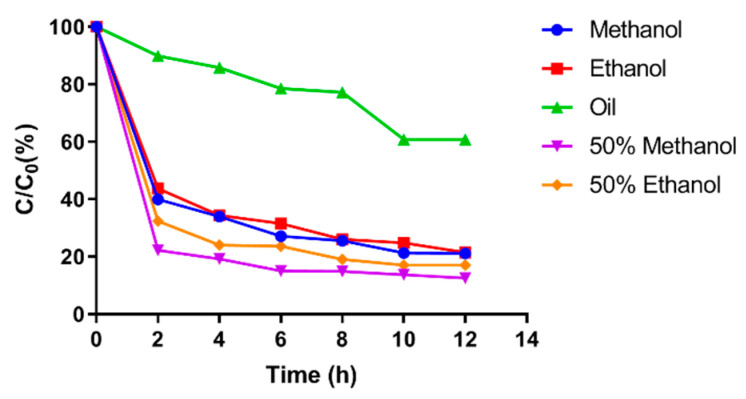
Degradation rate diagram of hydroxy-α-sanshool under UVB irradiation.

As depicted in Figure 6, the content of hydroxy-α-sanshool in methanol, anhydrous ethanol, soybean oil, 50% methanol, and 50% ethanol showed a continuous decrease with increasing time of UVB irradiation, with rapid degradation in the range of 0–10 h and a leveling off of the degradation rate at 10–12 h. The variation in the content of hydroxy-α-sanshool in soybean oil was relatively small compared with that in the remaining four solvents. The aforementioned results indicated that the degradation rate of hydroxy-α-sanshool increased continuously with increasing time of UVB irradiation under the same solvent conditions. UVB irradiation was more likely to cause the degradation of hydroxy-α-sanshool. However, soybean oil could maintain the stability of hydroxy-α-sanshool under UVB irradiation compared with the remaining four solvents.

**Figure 7:**
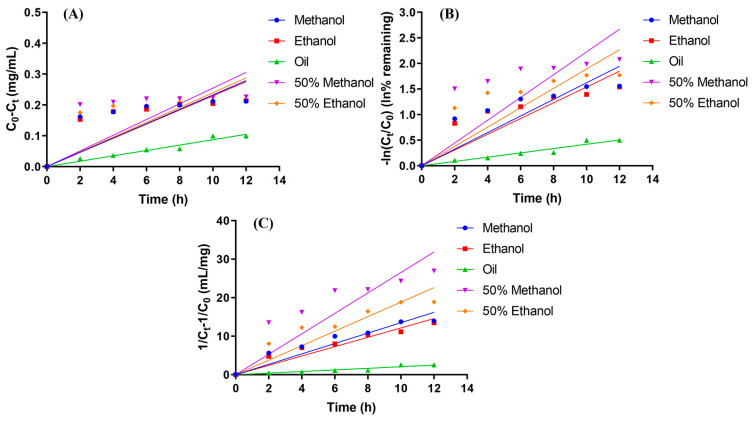
Kinetic fitting curve ((**A**) zero-order, (**B**) first-order, and (**C**) second-order reactions).

The content and degradation times of hydroxy-α-sanshool in different solvents were fitted with the kinetic equations of zero, one, and two levels to obtain the corresponding degradation kinetic curves and kinetic equations. The kinetic curves of different levels are shown in Figure 7, and their kinetic equations, degradation rate constants, and correlation coefficient results are presented in Table 5, Table 6 and Table 7.

As presented in Table 5, Table 6 and Table 7, a comparison of the correlation coefficients corresponding to different levels revealed that the degradation of hydroxy-α-sanshool in the rest of the solvents was better with the correlation coefficient *R*^2^ fitted using the secondary kinetic equation, except for the kinetic equation of hydroxy-α-sanshool degradation in soybean oil which tended to be of zero level. The correlation coefficient of the secondary kinetic equations in methanol, ethanol, 50% methanol, and 50% ethanol was 0.965, 0.972, 0.930, and 0.950, respectively, all of which were greater than 0.90. The kinetic equations fitted with the secondary kinetic equations for the degradation of hydroxy-α-sanshool were plotted with the vertical coordinate 1/C_t_–1/C_0_, which showed a linear relationship with the kinetic curve of UVB photodegradation time. This indicated that the kinetic process of UVB photodegradation of hydroxy-α-sanshool was more consistent with the second-order reaction.

The cuvette used in the experiment was made of quartz, which had a relatively high quantum efficiency and could enable the photoreaction of *Zanthoxylum* alkylamides more easily [33]. UV radiation is categorized into three main wavelength bands: UVA, UVB, and UVC. UVC has a relatively weak penetrating capacity and is used in a non-thermal method of disinfection. This study mainly focused on investigating the effects of UVB on the stability of *Zanthoxylum* alkylamides. It was found that the degradation kinetic process of hydroxy-α-sanshool was in line with the second-order reaction under UVB irradiation. The two methods of photo-oxidation and photocatalysis might occur directly under UVB irradiation compared with UVA irradiation, which had a shorter wavelength and higher photometric energy [34,35,36,37,38,39]. Therefore, the degradation of hydroxy-α-sanshool was more likely to occur under UVB irradiation compared to UVA irradiation. UVB irradiation was more detrimental to the stability of hydroxy-α-sanshool; therefore, avoiding UVB irradiation could improve its stability.

### 3.6. Analysis of UV Light Degradation Products and Degradation Pathways of Zanthoxylum Alkylamides

#### 3.6.1. HPLC Detection of UVB Light Degradation Products of *Zanthoxylum* Alkylamides

**Figure 8:**
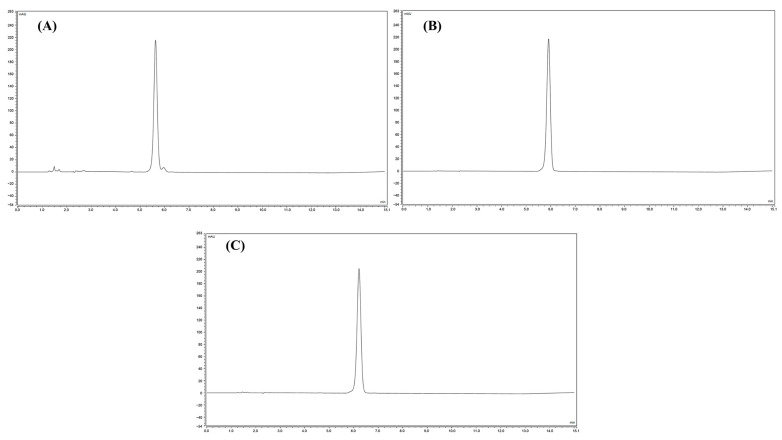
Liquid chromatograms of three sanshools ((**A**) hydroxy-ε-sanshool; (**B**) hydroxy-α-sanshool; (**C**) hydroxy-β-sanshool).

As shown in Figure 8, hydroxy-ε-sanshool, hydroxy-α-sanshool, and hydroxy-β-sanshool monomers purified with preparative liquid chromatography showed peaks at the corresponding retention times, and the order of their peaks was hydroxy-ε-sanshool, hydroxy-α-sanshool, and hydroxy-β-sanshool, respectively. The aforementioned chromatograms showed that the three purified sanshools had high purity.

**Figure 9:**
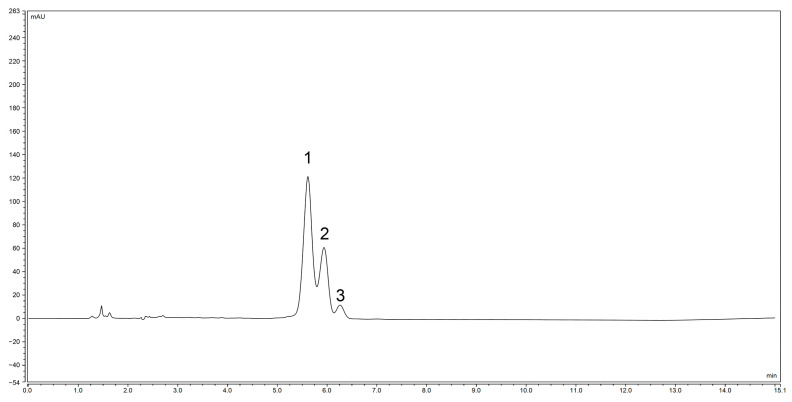
Liquid chromatogram of hydroxy-ε-sanshool exposed to UVB for 30 min (1. hydroxy-ε-sanshool; 2. hydroxy-α-sanshool; 3. hydroxy-β-sanshool).

**Figure 10:**
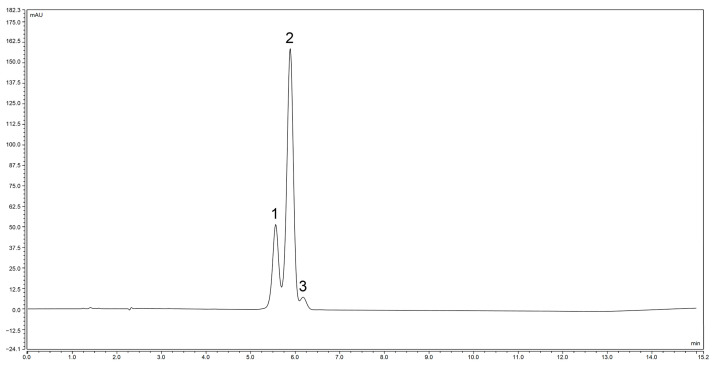
Liquid chromatogram of hydroxy-α-sanshool exposed to UVB for 30 min (1. hydroxy-ε-sanshool; 2. hydroxy-α-sanshool; 3. hydroxy-β-sanshool).

**Figure 11:**
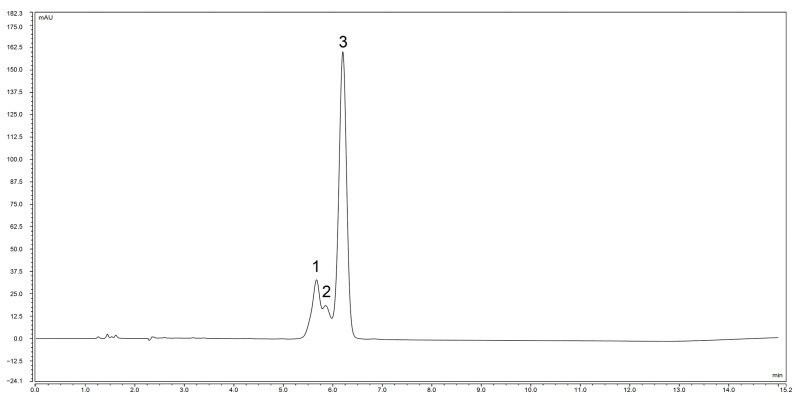
Liquid chromatogram of hydroxy-β-sanshool exposed to UVB for 30 min (1. hydroxy-ε-sanshool; 2. hydroxy-α-sanshool; 3. hydroxy-β-sanshool).

As shown in Figure 9, Figure 10 and Figure 11, the contents of the three monomeric sanshools, hydroxy-ε-sanshool, hydroxy-α-sanshool, and hydroxy-β-sanshool, were reduced after 30 min of UVB irradiation. Moreover, the other two sanshools were obtained from one sanshool under UVB irradiation, and the relative contents of the three sanshools in each group of samples were different. The aforementioned results indicated that all three sanshools, which constituted the *Zanthoxylum* alkylamides, could be transformed under UVB irradiation.

**Figure 12:**
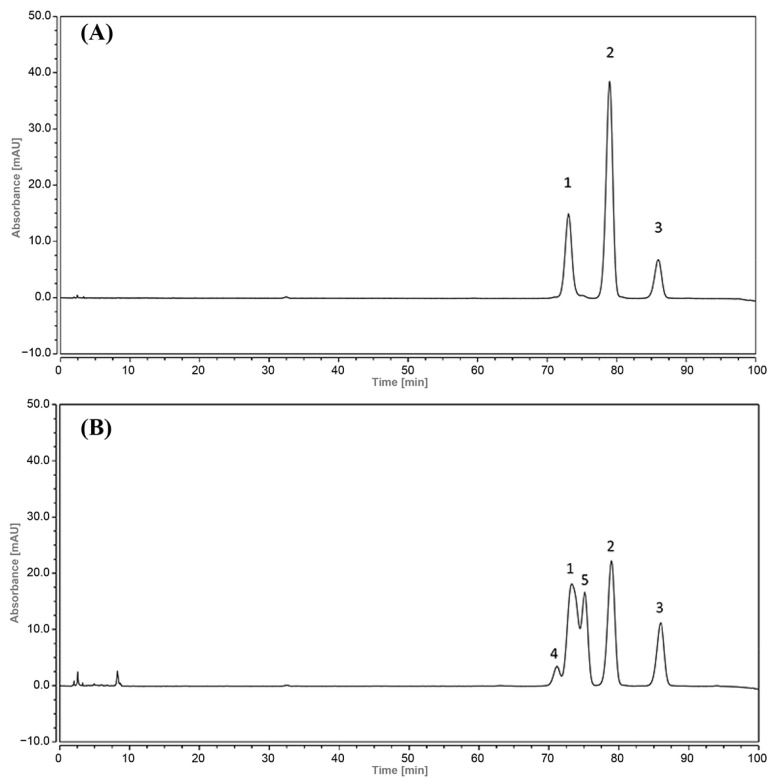
Chromatogram of *Zanthoxylum* alkylamides exposed to UVB for 30 min ((**A**) before uvb irradiation; (**B**) after UVB irradiation; 1. hydroxy-ε-sanshool; 2. hydroxy-α-sanshool; 3. hydroxy-β-sanshool; 4. degradation product 1; 5. degradation product 2).

By analyzing and comparing the chromatograms depicted in Figure 9, Figure 10 and Figure 11, it was found that the separation of *Zanthoxylum* alkylamides after UVB irradiation was not satisfactory. Therefore, the mixture of *Zanthoxylum* alkylamides after UVB irradiation for 30 min was analyzed with the HPLC method described in Section 2.6.2. As shown in Figure 12, the separation of the mixture of *Zanthoxylum* alkylamides was better when the new HPLC method was used, and two peaks appeared before and after the peak of hydroxyl-ε-sanshool. Therefore, it was presumed that the aforementioned two substances might be the degradation products of the numbing substances in *Z. bungeanum* after UVB irradiation.

#### 3.6.2. Preparative Liquid Chromatographic Separation and Purification of the UVB-Irradiated Degradation Products of *Zanthoxylum* Alkylamides

**Figure 13:**
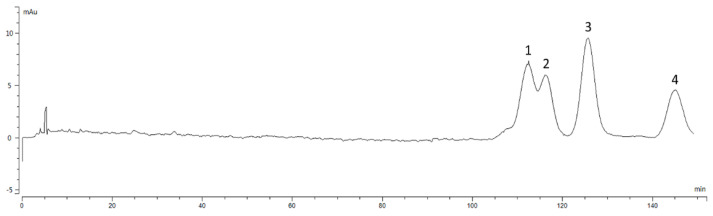
Preparative liquid chromatogram of *Zanthoxylum* alkylamides exposed to UVB (1. Hydroxy-ε-sanshool; 2. Degradation product; 3. Hydroxy-α-sanshool; 4. Hydroxy-β-sanshool).

As shown in Figure 13, the preparative column was a C18 column. The peak area of the degradation products before the retention of the hydroxy-ε-sanshool peak was small and poorly separated under the selected preparative liquid-phase conditions of methanol–water as the mobile phase, which was not convenient for the separation and purification with preparative liquid chromatography. Therefore, the degradation products after the retention of the hydroxy-ε-sanshool peak were considered for collection with preparative liquid chromatography and subsequent experimental studies.

#### 3.6.3. HPLC-MS Analysis of UVB Light Degradation Products of *Zanthoxylum* Alkylamides

**Figure 14:**
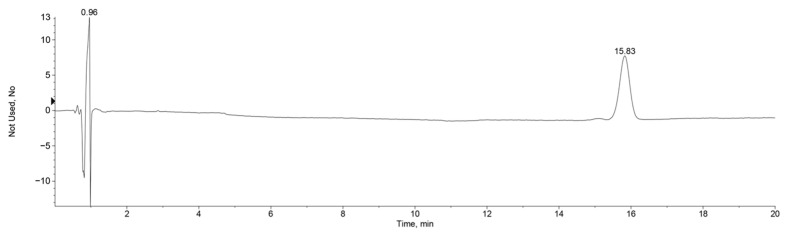
HPLC chromatogram of UVB degradation product.

**Figure 15:**
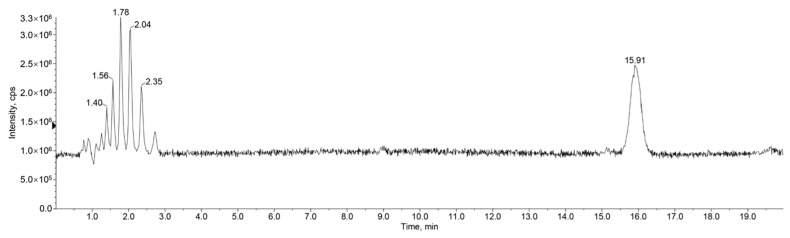
Total ion chromatograms of UVB degradation product.

**Figure 16:**
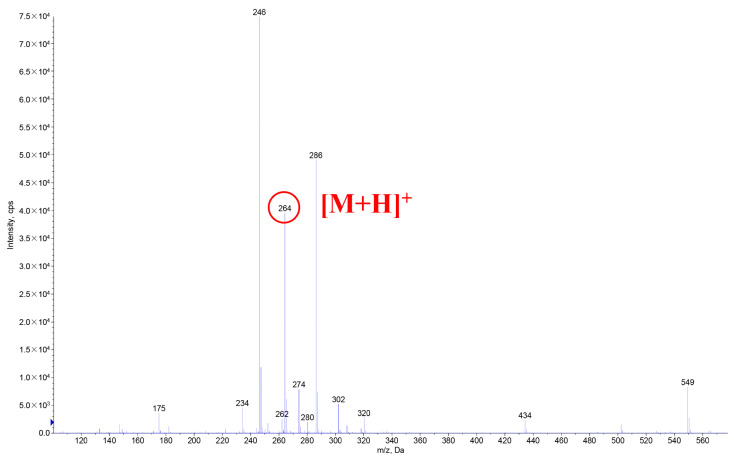
Mass spectra of UVB degradation product.

The HPLC chromatogram of UV degradation products in Figure 14 and the total ion flow diagram of HPLC-MS in Figure 15 show that the UVB degradation products obtained after the separation and purification with preparative liquid chromatography were of high purity and could be detected under the established HPLC-MS detection conditions. The mass spectra of the UVB degradation products in Figure 16 showed that the degradation products also had mass spectral peaks with *m*/*z* 264. The retention times of the HPLC peaks of the degradation products were relatively close to those of hydroxy-ε-sanshool and hydroxy-α-sanshool (Figure 12), and the separation was not high. Hence, it was speculated that the degradation products might be isomers of each other with three known sanshools with the molecular formula C_16_H_25_NO_2_ and a molecular weight of 263.

#### 3.6.4. Nuclear Magnetic Resonance H-Spectrum Analysis of UVB Degradation Products of *Zanthoxylum* Alkylamides

**Figure 17:**
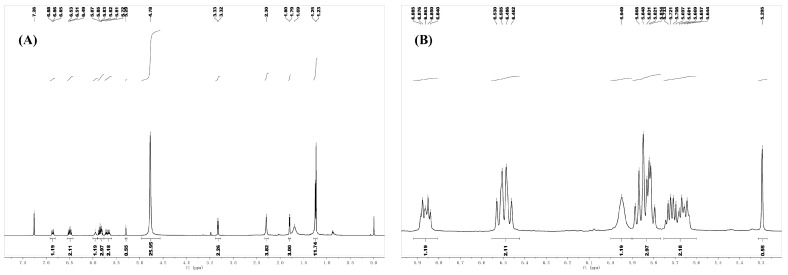
^1^H NMR spectrum of UVB degradation product ((**A**) full scan ^1^H NMR spectrum; (**B**) low-field ^1^H NMR spectrum).

**Figure 18:**
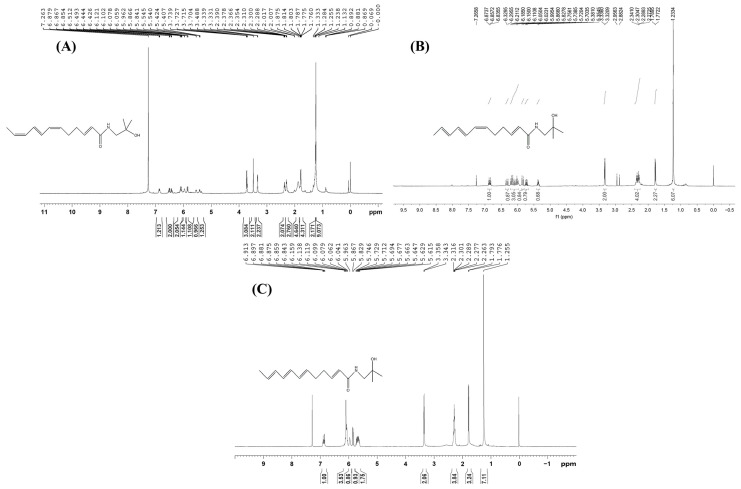
^1^H NMR spectrum of three sanshools ((**A**) hydroxy-ε-sanshool; (**B**) hydroxy-α-sanshool; (**C**) hydroxy-β-sanshool).

As shown in Figure 17, the NMR spectra of UVB degradation products were ^1^H NMR (DCCl3, 600 MHz): δ [ppm] 6.86 (dt, 1H, *J *= 15.6 Hz, 6.0 Hz), 6.49 (dd, 2H, *J* = 25.8, 14.4 Hz), 5.95 (s, 1H), 5.86–5.81 (m, 3H), 5.70 (dq, 1H, *J* = 14.4 Hz, 7.2 Hz), 5.66 (dt, 1H, *J* = 14.4 Hz, 7.2 Hz), 3.33 (d, 2H, *J *= 6.0 Hz), 2.29 (m, 4H), 1.80 (d, 3H, *J* = 7.2 Hz), and 1.23 (s, 6H). First, the NMR hydrogen spectra of this UV degradation product, especially the chemical shifts in the low field (i.e., the chemical shifts where the double-bonded hydrogen was located), were different from those of all three known substrates (Figure 18). However, the chemical shifts in the high field were consistent with those of the three known substrates, suggesting that the double-bonded conformation of the UVB degradation product might have changed, but the chemical structure of the high-field part was not.

**Figure 19:**
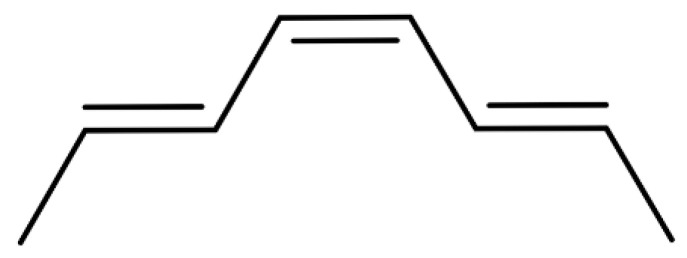
Similar structures in references.

**Figure 20:**
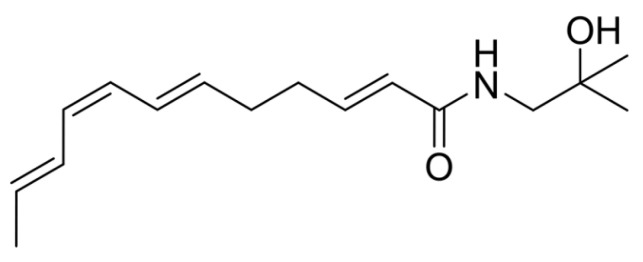
Structure of UVB degradation product.

By calculating the coupling constants between individual hydrogens and comparing them with similar structures in the published literature [40] (Figure 19), it was found that the double-bonded chemical shifts of the UVB degradation products matched with those reported in the published literature, that is, the double-bonded conformations in the UVB degradation products were *E*, *Z*, and *E*. Thus, the structure of the UVB degradation product of the numbing substances in *Z. bungeanum* could be inferred (Figure 20), which was named as (2*E*,6*E*,8*Z*,10*E*)-*N*-(2-hydroxy-2-methylpropyl)-2,6,8,10-tetraene-dodecanamide.

#### 3.6.5. Testing of UVB Degradation Products

**Figure 21:**
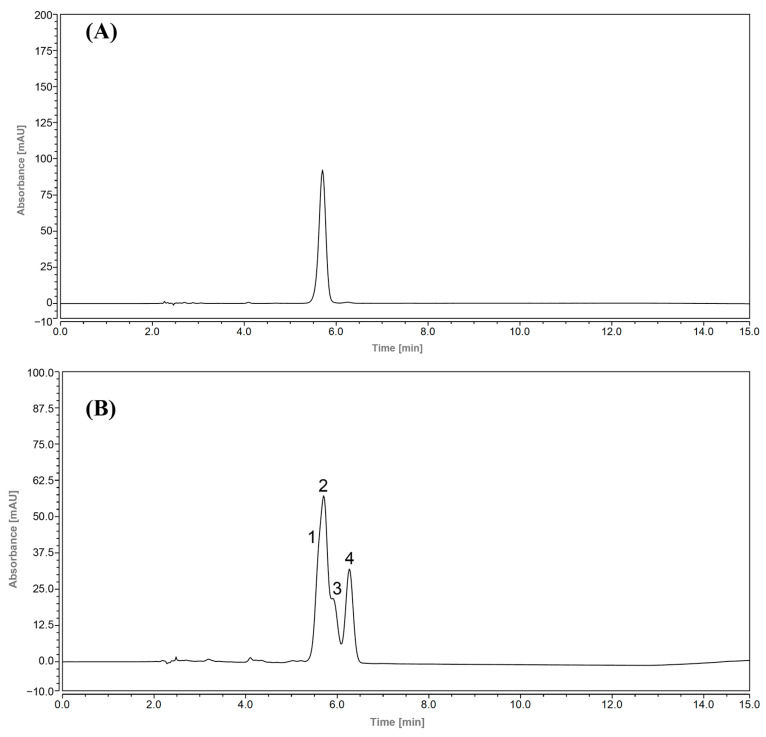
Liquid chromatogram of UVB degradation product exposed to UVB for 30 min ((**A**) before UVB irradiation; (**B**) after UVB irradiation; 1. hydroxy-ε-sanshool; 2. UVB degradation product; 3. hydroxy-α-sanshool; 4. hydroxy-β-sanshool).

As shown in Figure 21, the degradation also occurred after 30 min of UVB irradiation. The degradation products were mainly hydroxy-ε-sanshool, hydroxy-α-sanshool, and hydroxy-β-sanshool. Thus, it was observed that the aforementioned substances, tautomers, underwent isomerization under UVB irradiation, resulting in the interconversion between the sanshool structures.

### 3.7. UVB Light Degradation Pathways of Zanthoxylum Alkylamides

A correlation was found between the wavelength of UV light and the degradation of organic substances; the shorter the wavelength of UV light, the more it caused the photo-oxidation of the substance [38,39]. *Z* (“cis”) and *E* (“trans”) are geometric isomers, with differences in their physicochemical properties. For example, in the infrared spectrum, differences were found in terms of their C=C stretching vibrations, coupling constants in NMR, and so forth. Additionally, in the UV spectrum, the cis structure is susceptible to a blueshift in the absorption band due to the spatial effect, which affects its conjugation effect [41,42,43,44].

**Figure 22:**
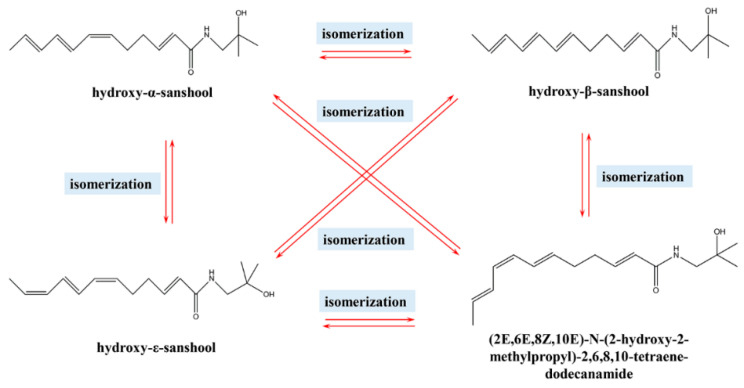
UVB degradation pathway of *Zanthoxylum* alkylamides.

The HPLC chromatograms, mass spectra, and NMR hydrogen spectra of these four substances, including hydroxy-ε-sanshool, hydroxy-α-sanshool, hydroxy-β-sanshool, and the isolated and purified UVB degradation products, were compared before and after UVB irradiation. The isomerization of the conjugated double-bonded part of the chemical structure might occur under an intensity of UVB irradiation, leading to the interconversion of chemical structures (Figure 22). This may be due to the isomer absorbing a certain amount of energy, thereby overcoming the binding force of the p orbital and rotating around the C-C σ-bond (through a semi-twisted transition state), which transforms from a *Z*-type to *E*-type isomer, or from an *E*-type to *Z*-type [45]. The photochemical processes of substances resulted in photochemical oxidation via the electron excitation of substances to produce radicals with homolytic cleavage or the transformation of a singlet oxygen from a triplet state to a triplet state molecular oxygen via energy transfer [46]. Therefore, in the experiments, not only was UV illumination found to cause structural transformation of the substances but the content of the substances was also reduced. However, the analysis of all UV degradation products of *Zanthoxylum* alkylamides is still difficult under the existing research conditions, and should be conducted in depth in the future.

## 4. Conclusions

In this study, *Zanthoxylum* oleoresin was used as the raw material. The monomeric components were isolated and purified, the changes in the content of *Zanthoxylum* alkylamides in different solvents were investigated under UVB conditions, and the degradation kinetic model equations were established. The results showed that the three substances isolated were hydroxy-ε-sanshool, hydroxy-α-sanshool, and hydroxy-β-sanshool. The degradation of *Zanthoxylum* alkylamides with UVB was more obvious, and the degradation reactions of hydroxy-α-sanshool in different solvents were second-order reactions. Additionally, the UVB degradation products were separated and purified using preparative liquid chromatography. Also, the UVB degradation pathways were explored with liquid chromatography, nuclear magnetic resonance, and liquid chromatography–mass spectrometry. The results showed that the three sanshools could generate new products, such as (2*E*,6*E*,8*Z*,10*E*)-*N*-(2-hydroxy-2-methylpropyl)-2,6,8,10-tetraene-dodecanamide, under UVB irradiation. The three sanshools and the products were isomers of each other. Each sanshool could undergo an isomerization reaction. In summary, the composition and content of *Zanthoxylum* alkylamides changed under UVB irradiation, which mainly occurred due to isomerization reactions. This study provided a theoretical reference for the effects of UVB irradiation on *Zanthoxylum* alkylamides and their products during storage. It also laid the foundation for the effective utilization of *Zanthoxylum* alkylamides.

## Figures and Tables

**Table 1:** Standard curve and correlation coefficient of *Zanthoxylum* alkylamides.

Samples	Standard Curve	Correlation Coefficient (*R*^2^)
*Zanthoxylum* alkylamides	Y = 120.99X − 4.3988	0.987
Hydroxy-ε-sanshool	Y = 49.776X + 0.1358	0.991
Hydroxy-α-sanshool	Y = 124.7X − 3.9475	0.987
Hydroxy-β-sanshool	Y = 129.64X − 0.2426	0.990

**Table 2:** Precision of the instrument.

Samples	Concentration (mg/mL)	RSD (%)
*Zanthoxylum* alkylamides	0.36	0.04
Hydroxy-ε-sanshool	0.02	1.3
Hydroxy-α-sanshool	0.32	0.06
Hydroxy-β-sanshool	0.02	0.3
*Zanthoxylum* alkylamides	0.7	0.09
Hydroxy-ε-sanshool	0.04	0.9
Hydroxy-α-sanshool	0.62	0.1
Hydroxy-β-sanshool	0.04	0.1
*Zanthoxylum* alkylamides	1.0	0.08
Hydroxy-ε-sanshool	0.06	1.2
Hydroxy-α-sanshool	0.88	0.09
Hydroxy-β-sanshool	0.06	0.3

**Table 3:** Determination of intraday and interday repeatability of *Zanthoxylum* alkylamides with HPLC.

Samples	Concentration (mg/mL)	RSD (%)	
		Intraday	Interday
*Zanthoxylum* alkylamides	0.36	0.75	0.72
Hydroxy-ε-sanshool	0.02	5.25	1.32
Hydroxy-α-sanshool	0.32	0.36	0.64
Hydroxy-β-sanshool	0.02	4.47	1.86
*Zanthoxylum* alkylamides	0.7	0.03	0.48
Hydroxy-ε-sanshool	0.04	1.72	3.01
Hydroxy-α-sanshool	0.62	0.03	0.27
Hydroxy-β-sanshool	0.04	0.14	2.34
*Zanthoxylum* alkylamides	1.0	0.11	0.31
Hydroxy-ε-sanshool	0.06	1.17	3.48
Hydroxy-α-sanshool	0.88	0.10	0.35
Hydroxy-β-sanshool	0.06	0.26	0.43

**Table 4:** Recovery of *Zanthoxylum* alkylamides.

Samples	Concentration (mg/mL)	RSD (%)
*Zanthoxylum* alkylamides	0.15	94.0
Hydroxy-ε-sanshool	0.01	89.8
Hydroxy-α-sanshool	0.13	93.0
Hydroxy-β-sanshool	0.01	110.0
*Zanthoxylum* alkylamides	0.30	102.8
Hydroxy-ε-sanshool	0.02	115.7
Hydroxy-α-sanshool	0.26	101.2
Hydroxy-β-sanshool	0.02	110.0
*Zanthoxylum* alkylamides	0.50	100.1
Hydroxy-ε-sanshool	0.03	104.3
Hydroxy-α-sanshool	0.44	99.1
Hydroxy-β-sanshool	0.03	116.4

**Table 5:** The fitting results of zero-order kinetic fitting curve of hydroxy-α-sanshool.

Solvent	Kinetic Equation	Degradation Rate Constant *k* (mg·mL^−1^·h^−1^)	Correlation Coefficient (*R*^2^)
methanol	C_t_ = 0.268 − 0.023*t*	0.023	0.852
ethanol	C_t_ = 0.272 − 0.023*t*	0.023	0.860
soybean oil	C_t_ = 0.254 − 0.009*t*	0.009	0.984
50% methanol	C_t_ = 0.259 − 0.025*t*	0.025	0.809
50% ethanol	C_t_ = 0.259 − 0.024*t*	0.024	0.831

Note: the rate constant *k* is the absolute value of the slope of the degradation kinetic equation, C_0_ is the content of hydroxy-α-sanshool before degradation, C_t_ is the content of hydroxy-α-sanshool after degradation, and *t* is the photodegradation time (h).

**Table 6:** The fitting results of first-order kinetic fitting curve of hydroxy-α-sanshool.

Solvent	Kinetic Equation	Degradation Rate Constant *k* (h^−1^)	Correlation Coefficient (*R*^2^)
methanol	ln(C_t_/C_0_) = −0.162*t*	0.162	0.909
ethanol	ln(C_t_/C_0_) = −0.154*t*	0.154	0.918
soybean oil	ln(C_t_/C_0_) = −0.042*t*	0.042	0.977
50% methanol	ln(C_t_/C_0_) = −0.222*t*	0.222	0.861
50% ethanol	ln(C_t_/C_0_) = −0.189*t*	0.189	0.887

Note: the rate constant *k* is the absolute value of the slope of the degradation kinetic equation, C_0_ is the content of hydroxy-α-sanshool before degradation, C_t_ is the content of hydroxy-α-sanshool after degradation, and *t* is the photodegradation time (h).

**Table 7:** The fitting results of second-order kinetic fitting curve of hydroxy-α-sanshool.

Solvent	Kinetic Equation	Degradation Rate Constant *k* (mg^−1^·mL·h^−1^)	Correlation Coefficient (*R*^2^)
methanol	1/C_t_ = 1.351*t *+ 1/0.268	1.351	0.965
ethanol	1/C_t_ = 1.214*t *+ 1/0.272	1.214	0.972
soybean oil	1/C_t_ = 0.207*t *+ 1/0.254	0.207	0.962
50% methanol	1/C_t_ = 2.655*t *+ 1/0.259	2.655	0.930
50% ethanol	1/C_t_ = 1.885*t *+ 1/0.259	1.885	0.950

Note: the rate constant *k* is the absolute value of the slope of the degradation kinetic equation, C_0_ is the content of hydroxy-α-sanshool before degradation, C_t_ is the content of hydroxy-α-sanshool after degradation, and *t* is the photodegradation time (h).

## Data Availability

Data are contained within the article.
